# Referral rate, profile and degree of control of patients with familial hypercholesterolemia: data from a single lipid unit from a Mediterranean area

**DOI:** 10.1186/s12944-023-01815-1

**Published:** 2023-05-11

**Authors:** Enric Serra-Planas

**Affiliations:** 1 Department of Endocrinology, Internal Medicine Service, Hospital Universitari d’Igualada, Consorci Sanitari de l’Anoia, Avinguda de Catalunya, 11, Igualada, Barcelona 08700 Spain; 2 Unit of lipids and cardiovascular risk, University Hospital of Igualada, Barcelona, Spain

**Keywords:** Familial hypercholesterolemia, Dutch lipid clinic network score, Lipoprotein (a), Lipid Unit

## Abstract

**Background:**

The challenging rigorous management of hypercholesterolemia promotes referral to specialized units. This study explored the need, based on referral rate and cardiovascular (CV) risk factor control in patients evaluated for familial hypercholesterolemia (FH), for a lipid unit (LU).

**Methods:**

Over a four-year period, 340 referrals to our unit were analyzed to establish the lipid disorder referral rate. Moreover, 118 patients referred for potential FH during the period 2010–2018 (52.4 ± 13.9 years, 47.5% male, Caucasian, 26.3% obese, 33.1% smokers and 51.7% with some glycaemic alteration) were investigated. The Dutch Lipid Clinic Network (DLCN) score, type and dose of lipid-lowering drugs, lipid profile including lipoprotein (a) (Lp(a)) and the presence of plaques with carotid ultrasound (CU) were recorded.

**Results:**

Lipids represented 6.2% of referrals (38 patient-years) requiring a 2–3 h weekly monographic outpatient consultation. The potential FH sample displayed a DLCN score ≥ 6 in 78% and modifiable CV risk factors in 51%. Only 22% achieved tight disease control despite intensive treatment. The statin-ezetimibe combination treatment group achieved better goals (73.0% vs. 45.5%, *P* = 0.003), and the rosuvastatin group had a higher proportion of prediabetes (60.9% vs. 39.1%, *P* = 0.037). Neither CU plaque presence nor Lp(a) > 50 mg/dL was linked with established CV disease patients, but higher Lp(a) concentrations were detected between them (102.5 (26.3–145.8) vs. 25.0 (13.0–52.0) mg/dL, *P* = 0.012).

**Conclusions:**

The referral rate, degree of control, and proportion of modifiable CV risk factors in FH patients demonstrate the need for LU in our area as well as optimize control and treatment.

## Introduction

Functional lipid units (LUs) began their activity in 1962 in Glasgow, and they are currently an essential part of the healthcare system [1]. In Spain, the main cause of referral to these LU is the study of genetic hypercholesterolemia, and the most common among them is familial hypercholesterolemia (FH) [2].

Different prevalence studies quantify that the heterozygous form affects 1:250–500 inhabitants, while the homozygous form is much less frequent at 1:1.000.000 [3–5]. In Denmark, a prevalence of 1 in 137 inhabitants has been estimated [4], in London 1:311 [6] and in our territory, Catalonia, it has recently been calculated that the population of patients with FH could reach 30.000 subjects or 1 in 256. Nevertheless, only 13% are identified and registered [7, 8]. For this reason, it may be stated that FH is underdiagnosed, and the existence of a LU as well as public or hospital cholesterol screening may be useful to achieve more and earlier diagnoses [9].

Due to an autosomal dominant genetic disorder, which predominantly affects the low-density lipoprotein cholesterol (LDLc) receptor gene (more than 1700 mutations have been described), although it can also affect the apolipoprotein B (apoB)-100 gene (LDLc receptor ligand) or the PCSK9 gene (which increases LDLc receptor degradation), the process of LDLc degradation changes. Subsequently, this disease presents high concentrations of LDLc in serum and an accumulation of these particles in the tissues with consequent premature atherosclerosis and coronary artery disease (CAD) [10, 11].

For its diagnosis, there exist different scales that stratify the probability, such as the Make Early Diagnosis to Prevent Early Death (MEDPED) [12] by the National Lipid Association of the United States, the Simon Broome scale [13], which is used in the United Kingdom and, evaluated in our territory [14], the Dutch Lipid Clinic Network (DLCN) score [15]. These diagnostic tools have many points in common since they assign a specific risk to certain personal, familial, clinical and laboratory parameters. Among them and according to studies in the Spanish population, the DLCN approach presents greater sensitivity and specificity for the diagnosis of FH, although its calculation requires all the information to be available [14, 16].

The importance of detecting subjects affected by FH lies in the fact that they have a higher risk of presenting CAD, up to ten times higher, than the general population despite cholesterol treatment [4, 17]. For this reason, multidisciplinary LU is an essential health tool for diagnosis, risk stratification, lipid-lowering treatment adaptation for these patients and screening FH in first-degree relatives.

The objectives of this study were to evaluate the referral rate for lipid management from primary health care and to define the profile of patients evaluated and treated for FH in our outpatient clinics during the last eight years to demonstrate the need to create a LU to respond to this demand and to adapt preventive and specific therapeutic strategies for patients with FH from our territory.

## Methods

### Referrals and patients

#### Analysis of the referral rate for the study of lipid disorders to the Endocrinology unit

The sample size for evaluating the percentage of referrals to the Endocrinology Outpatient Consultation for lipid disorders was calculated using the GRANMO sample size calculator from IMIM (Hospital del Mar Medical Research Institute) version 7.12, April 2012.

Following a population proportion estimation model, a minimum sample size of 340 subjects was estimated according to a percentage of referral to specialized consultation for lipid disorders of 14.7% [18], accepting an alpha error of 0.05, a beta error of 0.2, and a unilateral contrast model, assuming a loss percentage of 1% and a minimum difference to be detected of 0.05% units.

Following a retrospective observational study model, the main reason for every referral from primary care to the Endocrinology Unit of our center during the period 01/2013 to 12/2016 was recorded until a representative sample of 340 referrals was obtained.

#### Analysis of the baseline characteristics of patients referred for the study of familial hypercholesterolemia

Using a similar process and among those patients attended in the endocrinology outpatient clinic from 01/2010 to 12/2018 referred for the study of a possible FH, it was calculated that a sample of 116 individuals would allow us to estimate their main characteristics (with a confidence of 95%, a precision of ± 1% and assuming 10% loss).

An initial cohort of 138 patients was enrolled in this cross-sectional study. The established inclusion criteria were being older than 18 years and having been evaluated previously on suspicion of FH, in its heterozygous form, from 01/2010 to 12/2018 in our unit. The exclusion criteria for the study were not fulfilling the inclusion criteria and missing essential data for DLCN score calculation. Twenty patients were excluded due to a lack of information.

As part of the FH follow-up, a high percentage of these patients were evaluated with carotid ultrasonography (CU) for the detection of plaques and with the measurement of lipoprotein(a) (Lp(a)).

Age at diagnosis and current age, sex, weight, height and body mass index (BMI) were recorded in all patients and included in the study as quantitative variables. Waist circumference was collected in 60% of the sample. Characterization into overweight, obesity and central obesity was established following the cutoff points defined by the IDF (International Diabetes Federation) and SEEDO (Spanish Society for the Study of Obesity) [19, 20] and diabetes or prediabetes condition according to ADA (American Diabetes Association) diagnostic criteria [21]. However, to compare with local studies, prediabetes was also established following the guidelines of the SED (Spanish Diabetes Society) working group [22].

The DLCN score was evaluated for each patient, and as qualitative variables, obesity, smoking, the use of statins (establishing the type and dose) and other lipid-lowering drugs were recorded as well as the performance of the genetic study and the mutation detected. Active smokers and ex-smokers who had been quit for less than 5 years were considered the same category in relation to residual cardiovascular (CV) risk [23].

### Diagnostic criteria

#### Familial hypercholesterolaemia

For the aforementioned reasons, the probability of diagnosis of FH was made using the DLCN scale, which establishes a numerical value for family, personal, pathological and analytical history of the patients and sums the different results in a final score. The identification of a mutation affecting the LDLc receptor gene, apoB or PSCK9 is associated with 8 points [15, 24].

Each patient was stratified into a diagnosis of certainty (> 8 points), probability (6–8 points) and possibility (3–5 points). A score below 3 points was considered unlikely [15].

#### Established cardiovascular disease and control target

According to the 2019 ESC/EAS Guidelines for the management of dyslipidemia [25], we assumed established cardiovascular disease (CVD) in all patients with a history of acute coronary syndrome (myocardial infarction or unstable angina) or CAD, such as stable angina or coronary revascularization requirements. Moreover, transient ischemic attack, stroke and peripheral arterial disease were included.

The lipid control target, always based on the 2019 European guidelines [25], for patients at high and very high cardiovascular risk (where patients with FH are included) requires a reduction in LDLc of at least 50% from baseline and LDLc concentrations below 70 and 55 mg/dL, respectively.

#### Evaluation of subclinical atherosclerosis

Most patients were evaluated with the performance of a CU. Every study was performed by a trained radiologist to identify the presence of atheromatous plaques and establish their size. CU was not always performed by the same specialist due to the inclusion of patients over a period of 8 years.

An atheromatous plaque was considered as the detection of a thickening ≥ 1.5 mm, a focal lesion invading the lumen at least 0.5 mm or a lesion invading the lumen ≥ 50% of the surrounding carotid intima-media thickness. The images were recorded by a 13.5 M*Hz* linear transducer of a B-mode ultrasound machine (General Electric Company, LogiQ P9, manufactured in China, 2007).

### Biochemical measurements

Blood tests were obtained at approximately 8:00 a.m. after fasting for at least 8 h. The lipid profile was obtained from every patient, including LDLc by the Friedewald formula and total cholesterol, high-density lipoprotein (HDL), non-HDL cholesterol (total cholesterol - HDL) and triglycerides by routine procedures.

#### HbA1c

HbA1c was calculated in ethylenediaminetetraacetic acid blood samples by high-performance liquid chromatography using a fully automated analyzer (Adams Menarini HI-AUTO A1c 8160, from Arkray (Kyoto, Japan)), interassay coefficient of variation of 1.8 and 1.5% (HbA1c levels of 4.8 and 9.0%) and reference range of 4–6.1%. This test procedure is certified by the National Glycohemoglobin Standardization Program for traceability to the Diabetes Control and Complications Assay reference method.

#### Lipoprotein (a)

Lp(a) mass was determined in serum by latex-enhanced turbidimetric immunoassay (The Binding Site Optilite®) at the reference laboratory. A risk value of Lp(a) higher than 50 mg/dL was assumed in accordance with most clinical guidelines and the Caucasian nature of the study population [26].

### Statistical methods

Statistical package for the social sciences (IBM, SPSS, Chicago, IL, USA) for personal computers, version 19.0, was used for statistical analysis.

All continuous variables were recorded as the mean and standard deviation, except those with a nonnormal distribution, which were expressed as the mean and interquartile range. Instead, categorical variables were collected as frequencies or percentages.

To establish differences between groups, Student’s t test, the Mann‒Whitney U test (nonparametric) and the squared X^2^ test were used, assuming a *P* value of less than 0.05 as statistically significant.

## Results

### Referral rate for the study of lipid disorders to the Endocrinology Unit from primary care

Table 1 summarizes the main reasons for referral from primary care to the Endocrinology Unit during a four-year period 2013–2016.

**Table 1:** Referrals from primary care to the Endocrinology Unit during the period 01/2013–12/2016

***n*** **(n/%)**	340 (100%)
**Type 1/Type 2 diabetes (n/%)**	19/79 (5.6/23.2%)
**Thyroid nodules (n/%)**	62 (18.2%)
**Hyperthyroidism (n/%)**	60 (17.6%)
**Hypothyroidism (n/%)**	32 (9.4%)
**Morbid obesity (n/%)**	28 (8.2%)
**Lipid disorders (n/%)**	21 (6.2%)
**Hyperprolactinemia (n/%)**	13 (3.8%)
**Hyperandrogenism (n/%)**	6 (1.8%)
**Hypercortisolism (n/%)**	4 (1.2%)
**Hypogonadism (n/%)**	3 (0.9%)
**Hyperparathyroidism (n/%)**	3 (0.9%)
**Hypoglycemia (n/%)**	2 (0.6%)
**Hyperhidrosis (n/%)**	2 (0.6%)
**Gynecomastia (n/%)**	2 (0.6%)
**Others (n/%)**	4 (1.2%)

Percentage quantification of referrals to the Endocrinology Unit in the period 01/2013-12/2016, where the rate of referral for lipid abnormalities obtained sixth position in terms of frequency.

The evaluation of a representative sample of main referrals from primary care to our Endocrinology Unit of the University Hospital of Igualada showed that the study, treatment and management of lipid disorders were some of the main causes of referral, specifically the sixth position in volume, up to 6.2% of them. With approximately 38 cases referred for this reason per year, a referral rate of 33.9 × 10^5^ inhabitants can be estimated.

This information allowed us to calculate the number of hours of assistance required to attend to lipid disorder referrals to our unit, which resulted in a monographic unit for outpatient lipid care of approximately 2–3 h per week. For this calculation, we assumed an average time of first visits and subsequent visits of 20 and 15 min, respectively, a population of 112,107 inhabitants in our influence area, 240 days of medical assistance per year and an outpatient performance of 80% [27].

### Characterization of patients evaluated for familial hypercholesterolemia during the 2010–2018 period

Table 2 summarizes the main characteristics of the study sample.

**Table 2:** Main characteristics of study sample

***n*** **(n/%)**	118 (100%)
**Gender (M/F) (n/%)**	56 / 62 (47.5/52.5%)
**Age at diagnosis (years)**	33.9 ± 16.0
**Age (years)**	52.4 ± 13.9
**Weight (Kg)**	74.3 ± 15.5
**Height (Cm)**	165.4 ± 10.1
**BMI (Kg/m** ^**2**^ **)**	27.1 ± 4.8
**Waist circumference (M/F) (cm)**	99.5 ± 12.9 / 90.8 ± 13.2
**Obesity (n/%)**	31 (26.3%)
**Smoking habit (n/%)**	39 (33.1%)
**Hypertension (n/%)**	31 (26.3%)
**Diabetes (n/%)**	9 (7.6%)
**Prediabetes (n/%)**	52 (44.1%)
**Prediabetes regional criteria (n/%)**	26 (22.0%)
**CVD (n/%)**	23 (19.5%)
**Statin treatment (n/%)**	110 (93.2%)
**Ezetimibe treatment (n/%)**	75 (63.6%)
**Statin-Ezetimibe combination (n/%)**	74 (62.7%)
**iPSCK9 (n/%)**	6 (5.1%)

**Kg**: kilograms, **Cm**: centimeters, **BMI**: body mass index, **CVD**: cardiovascular disease, i**PSCK9**: proprotein convertase subtilisin/kexin type 9 inhibitors

#### Familial hypercholesterolemia diagnostic characteristics

The DLCN scale was conducted on all study participants as summarized in Table 3. Seventy-eight subjects displayed a DLCN score greater than or equal to 6 points as a probable or certain diagnosis of FH. From family background information, LDLc concentrations above 210 mg/dL followed by early ischemic heart disease in a first-degree relative were the most frequent antecedents (91 and 61 patients (77.1% and 51.7%, respectively). Similarly, early ischemic heart disease reached the highest proportion among personal history (17 patients (14.4%)). 8% displayed tendon xanthomata or arcus cornealis.

**Table 3:** Dutch Lipid Clinic Network items frequencies

Family history	*n* (%)	
First-degree relative with known premature (men aged < 55 years; women < 60 years) coronary or vascular disease (1 point)	61 (51.7%)	
First-degree relative with known LDL above the 95th percentile (1 point)	91 (77.1%)	
First-degree relative with tendinous xanthomata and/or arcus cornealis (2 points)	11 (9.3%)	
Children aged < 18 years with LDL above the 95th percentile (2 points)	50 (42.4%)	
**Clinical history**		
Patient with premature (men aged < 55 years; women < 60 years) coronary artery disease (2 points)	17 (14.4%)	
Patient with premature (men aged < 55 years; women < 60 years) cerebral or peripheral vascular disease (1 point)	5 (4.2%)	
**Physical examination**		
Tendinous xanthomata (6 points)	11 (9.3%)	
Arcus cornealis before age 45 years (4 points)	6 (5.1%)	
**LDL-C levels (without treatment)**		
LDL ≥ 8.5 mmol/L (≥ 330 mg/dL) (8 points)	8 (6.8%)	
LDL 6.5–8.4 mmol/L (250–329 mg/dL) (5 points)	42 (35.6%)	
LDL 5.0–6.4 mmol/L (190–249 mg/dL) (3 points)	41 (34.7%)	
LDL 4.0–4.9 mmol/L (155–189 mg/dL) (1 point)	22 (18.6%)	
LDL < 4.0 mmol/L (< 155 mg/dL) (0 point)	5 (4.2%)	
**DNA analysis**		
Functional mutation in the LDLr, apoB or PCSK9 genes (8 points)	33 (27.9%)	
**FH diagnosis**		
Definite FH (> 8 points)	51 (43.2%)	
Probable FH (6–8 points)	40 (33.9%)	
Possible FH (3–5 points)	23 (19.5%)	
Unlikely FH (0–2 points)	4 (3.4%)	

**LDL**: Low density lipoprotein cholesterol, **LDLr**: low-density lipoprotein receptor, **apoB**: Apolipoprotein B, **PCSK9**: proprotein convertase subtilisin/kexin type 9, **DNA**: deoxyribonucleic acid, **FH**: familial hypercholesterolaemia

Items of the DLCN scale for the diagnosis of FH including family history, clinical history, physical examination, pre-treatment LDL cholesterol concentrations, and genetic study.In the right column, number and percentage of patients of the study who presented the item.

Pretreatment LDLc concentrations above 250 mg/dL were detected in 50 patients, and genetic analysis was performed in 54 patients (45.8% of the sample), detecting a genetic mutation in 33 patients (63.5% of these cases analysed). The most frequent mutations detected were those that affected the LDLc receptor gene. In a single patient, a PSCK9 gene mutation was detected, whereas an apoB gene mutation was noticed in another subject.

Male patients demonstrated higher DLCN scores (8.5 (6.3–13.0) vs. 7 (5.0–11.3) in women, *P* = 0.026) and a higher percentage of certain FH diagnoses than females (38 (67.9%) vs. 18 (32.1%) among certain FH patients, *P* = 0.008).

#### Cardiovascular risk factor assessment

A high prevalence of a modifiable CV risk factor, such as a smoking habit, was detected. Thirty-nine patients were considered to be smokers (31 active smokers and 8 ex-smokers 5 years maximum smokefree). In the same way, a quarter of the sample presented obesity (≥ 30 kg/m^2^), and according to the information of 71 patients, abdominal obesity (defined as ≥ 94 cm in men, 80 in women) was established in 70.4% of this subgroup. In the group of patients with a probable or certain diagnosis, similar percentages were detected (31.5% smoking, 25% obesity and 66.7% central obesity). No differences were detected between sex and these or other CV factors, such as hypertension or diabetes.

Diabetes was present in 9 patients in the sample (7.6%). Concerning prediabetes, its proportion depended on the cutoff points adopted, detecting 52 or 26 patients among those without diabetes (44.1% or 22% using regional criteria). A higher proportion of patients with prediabetes was observed among those treated with rosuvastatin (60.9% vs. 39.1% in the nonrosuvastatin group, *P* = 0.037) and in those treated with statins other than simvastatin or high doses of atorvastatin (40–80 mg) (84.8% vs. 15.2% in the simvastatin/high-dose atorvastatin treatment group, *P* = 0.001), but this relationship was not dose-related in any of the cases. Patients with prediabetes were older (56.2 ± 13.9 vs. 47.5 ± 12.7 years, *P* = 0.002) and presented a greater waist circumference (96.9 ± 13.2 vs. 89.8 ± 12.7 cm, *P* = 0.047) than patients without prediabetes, although no significant differences were detected between gender, DLCN score, obesity, central obesity, BMI or statin dose between these two groups.

#### Subclinical atherosclerosis and cardiovascular disease

Approximately 20% of the study sample presented CVD: 17 subjects with early myocardial infarction (MI), 5 with early cerebral stroke and 1 subject with MI during follow-up in the unit.

CU and Lp(a) were performed in more than 70% and 55% of our study cohort, respectively. Specifically, 86 patients in the sample were evaluated for CU, 69 of whom were in primary prevention and 17 of whom were in secondary prevention (approximately 80–20% ratio).

Forty-two participants had carotid plaques (47.7%), and 32 showed Lp(a) concentrations above 50 mg/dL (31.3%) among those evaluated. Among patients with carotid plaques, a higher proportion of males was detected (64.3% vs. 35.7% in females, *P* = 0.018).

Patients with established CVD showed higher Lp(a) concentrations (102.5 (26.3–145.8) vs. 25.0 (13.0–52.0) mg/dL in those without CVD, *P* = 0.012), and if we considered the group of patients with established CVD together with the group of patients with subclinical CVD based on the detection of atheromatous plaques, an association with Lp(a) above 50 mg/dL was observed (15 (71.4%) vs. 6 (28.6%) among those without established or subclinical CVD, *P* = 0.05). However, separately, there was no association between established CVD and the detection of plaques by CU or Lp(a) concentrations above 50 mg/dL. There were no associations between CU or Lp(a) and DLCN score, smoking, hypertension, obesity, central obesity, diabetes or prediabetes.

#### Treatment, achievements and follow-up

More than 90% of the sample was under statin treatment, 48.3% with rosuvastatin (18.6% in maximum dose of 40 mg a day), 29.7% with atorvastatin (11% with 80 mg a day), 26.3% with intermediate or low intensity statins and 62.7% with statin-ezetimibe combination (Table 4). Only six patients were under treatment with iPSCK9, 3 patients were not achieving LDLc goals with statin treatment, and the others were treated for statin intolerance.

**Fig. 1:**
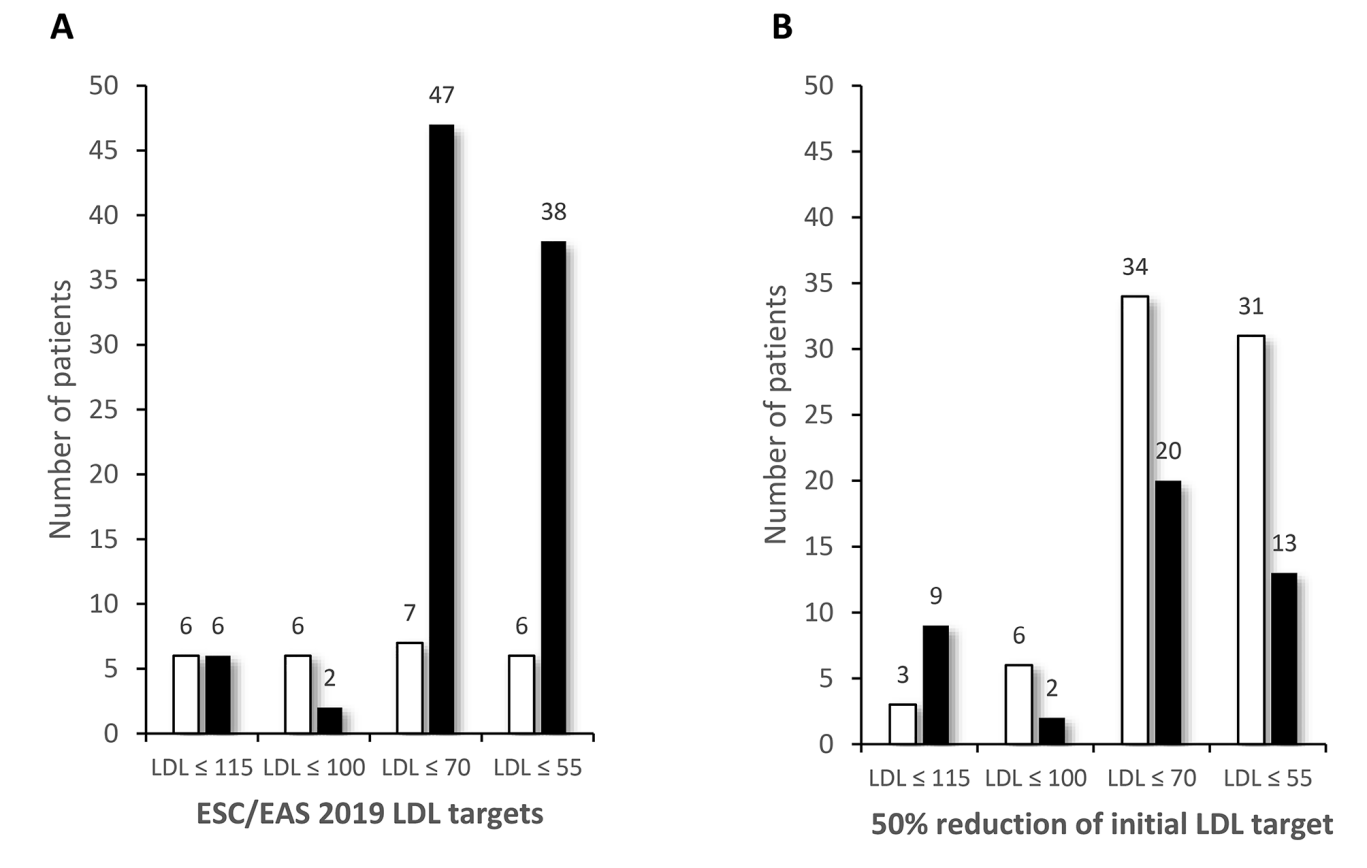
Achievement of control in LDL target groups. **Table A** ESC/EAS 2019 LDL targets. For each LDL control targets, white columns count the patients who achieved the LDL target and the black ones those who did not. **Table B** 50% reduction of initial LDL target. White columns count the patients who achieved the LDL target and the black ones those who did not

Only 22% of the sample achieved strict disease control when ESC/EAS 2019 goals were assumed [25]; this percentage increased to 62.7% if a 50% reduction from initial LDLc concentrations was considered (Fig. 1), and only 19 patients (16.1% of the sample) achieved both objectives.

**Table 4:** Treatment characteristics

Statin treatment		*n* (%)
**Rosuvastatin**		**57 (48.3)**
40 mg/day		22 (18.6)
20 mg/day		30 (25.4)
10 mg/day		5 (4.2)
**Atorvastatin**		**35 (29.7)**
80 mg/day		13 (11.0)
40 mg/day		14 (11.9)
20 mg/day		8 (6.8)
**Simvastatin**		**8 (6.8)**
40 mg/day		4 (3.4)
20 mg/day		3 (2.5)
10 mg/day		1 (0.8)
**Pitavastatin 4 mg/day**		**10 (8.5)**
**High intensity**		**79 (66.9)**
**Statin intolerance**		**8 (6.8)**
**Cholesterol absorption inhibitors**		
**Ezetimibe**		**75 (63.6)**
**Fibrate treatment**		
**Fenofibrate**		**6 (5.1)**
**Polyunsaturated fatty acids**		
**Omega-3**		**2 (1.7)**
**Dietary supplement**		
**Red yeast rice**		**2 (1.7)**
**PCSK9 inhibitors**		
**PSCK9 inhibitors**		**6 (5.1)**
**PSCK9**: proprotein convertase subtilisin/kexin type 9		

High intensity statins include rosuvastatin 20–40 mg a day and atorvastatin 40–80 mg a day.

Patients treated with statin-ezetimibe combinations showed a higher proportion, achieving the 50% target (73.0% vs. 45.5% in the no combination group, *P* = 0.003). This association was neither type- nor dose-dependent and was not observed in patients receiving treatment with statins alone or in combination with iPSCK9.

## Discussion

There were two main results derived from the present study. First, this investigation demonstrated that in our region in central Catalonia, the constitution of an LU was necessary to respond to referrals from primary care for the study, treatment, and management of lipid disorders.

The other main finding was the poor degree of control, with only 22% of the sample achieving lipid targets despite treatment with high-intensity and high-dose statins (69% and 30%, respectively) and the presence of 51% of modifiable CV factors in a high and very high CV risk cohort.

Regarding the referral rate, lipid profile alterations are the main CV risk factor in our country, and their prevalence rises to 49.3% in some series [28]. Unfortunately, adequate control is achieved in only one-fifth of patients [29, 30]. This situation, partly explained by an underdosage of statins [31, 32], justifies the creation and actualization of territorial LU.

Our study showed that referral for dyslipidemia from primary care is frequent, 6.2% of derivations (38 patient-years or a ratio of 33.9 × 100,000 inhabitants), but significantly lower than that reported by other authors [18, 33]. Even so, our referral rate is sufficient to require a weekly monographic unit for outpatient lipid care based on clinical management tools [27]. One possible explanation for this low referral rate could be related to the fact that the LU is part of the Endocrinology Unit, and these patients could have been referred to other services, such as internal medicine or cardiology. On the other hand, another potential explanation could be the lack of awareness of the existence of the LU, so its promotion should be a priority in this new period, in line with the recommendations of the SEA (Spanish Society of Arteriosclerosis) [1].

Regarding the characterization of patients with heterozygous FH in our area, the most frequent DLCN scale parameters were first-degree relatives with LDLc > 210 mg/dL among family history (91 patients (79.1%), early ischemic heart disease among personal history (17 patients (14.4%)) and LDLc > 250 mg/dL (50 patients (42.4%)). Among those with definite FH, there were more males (38 (67.9%) vs. 18 (32.1%), *P* = 0.008). These results are similar to those reported in Copenhagen [34], where a population of 69,016 subjects (4295 possible, 502 probable/definitive) was evaluated by DLCN. The probable/definitive group displayed higher percentages in the same variables (50 and 28%, respectively), and 58% of patients had LDLc > 250 mg/dL. In this study, no significant differences were detected in terms of gender, only after 79 years of age, which is attributed to the late onset of CAD in the female sex. This reason could also explain our findings.

Concerning lipid control, our results were in the same direction as those of the Froylan D MS study group [35], who evaluated 1196 Mexican patients with CAD (54 ± 8 median age, 80.3% men and 2.4 median years of evolution of CAD), and the percentage of LDLc under tight control was 11.4%. This group followed the American Association of Clinical Endocrinologists Guidelines [36] that established lipid goals below 55 mg/dL. Among our group of 44 patients with FH and very high CV risk (59.5 ± 10.5 median of age, 61.9% men) with the same strict lipid control goal, only 6 patients (13.6% of this group and 5% of the entire sample) achieved such a low concentration. In contrast to other authors, no association was detected between failure to achieve therapeutic goals and positive genetic studies [37].

Closer, in SpAnish Familial hypErcHolEsterolemiA cohoRT (SAFEHEART) [38], 4132 patients with heterozygous FH were evaluated (45.0 (34.0–56.0) median age, 45.5% men and a follow-up of 5.1 ± 3.1 years) following the guidelines of the European Atherosclerosis Society and American Heart Association from 2016 [39], which established a target of LDLc below 70 mg/dL for FH with very high CV risk, detected only 4 patients at inclusion and 13 at the follow-up (1.1 and 4.7% of the sample) who reached this goal.

The ezetimibe-statin combination group achieved better results in terms of LDLc and had a better percentage of LDLc target achievement, suggesting a greater reduction in CV risk [40]. Due to the small number of patients treated with iPSCK9 and the nature of the data analysed, it was not possible to demonstrate, although it is very likely, an improvement in lipid profile, adherence to treatment and quality of life compared to the other groups [41].

Regarding other CV risk factors, a high prevalence of those modifiable in patients with DLCN ≥ 6 could be translated as a failure of one of the main functions of the LU [1]. Percentages of 31.5% smoking, 25% obesity and 22.8% hypertension are equivalent to or worse than the overall prevalence in Spain [28], although this is a population with an increased risk of CAD between 10- and 13-fold [4] and risk of new cases of CVD, especially among those with an aggregation of CV risk factors [42].

In contrast to the diabetes ratio, a considerable proportion of patients with prediabetes, higher than that reported in the general population [28], was detected. This high percentage of prediabetes was probably related to the hyperglycemic effect of statins [43] and the low proportion of diabetes to the reduced mean age of the study sample. The highest proportion was identified among those treated with rosuvastatin and those who used statins other than simvastatin or high doses of atorvastatin, unlike other authors [44].

Focusing on the CV characterization of patients with FH, in our study, we observed a better association between Lp(a) concentrations and CVD than carotid plaque detection by CU. Those with established CVD displayed greater concentrations (102.5 (26.3–145.8) vs. 25.0 (13.0–52.0) mg/dL in those without CVD, p = 0.012), and those with established and/or subclinical CVD displayed a greater proportion of Lp(a) above 50 mg/dL (15 (71.4%) vs. 6 (28.6%) among those without established or subclinical CVD, p = 0.05). In the same direction, we find different risk equations, such as the Familial Hypercholesterolemia Risk Score (FH Risk Score) [45] or the SAFEHEART Registry (SAFEHEART-RE) [46], among others [47], which use Lp(a) concentrations but not ultrasound as a parameter to establish risk in this type of patient. Nevertheless, the small number of patients included in the study, as well as the partial percentage of CU performed, could explain the absence of a relationship between these variables.

### Study strengths and limitations

The cross-sectional design of the study, the application of the DLCN according to anamnesis or self-reference of the patients in reference to personal and family history, lack of information on waist circumference, adherence to therapy, CU or Lp(a) concentrations in some patients and the change in LDLc targets over the years were potential limitations of our observational study. Another weakness could be related to the variability in the detection of atheromatous plaques due to the participation of different radiology specialists.

## Conclusions

In summary, this study confirms the need for an LU in our territory based on the referral data from primary care, the poor degree of control of LDLc concentration and the inadequate proportion of modifiable CV risk factors in a population with an increased CV risk associated with FH. The creation and approval of the LU, as well as the adoption of new control objectives and follow-up parameters, could reduce the high CV risk of this group of patients in the future.

## Data Availability

The anonymized database can be accessed by contacting the corresponding author.

## List of abbreviations

LU: lipid units
FH: familial hypercholesterolemia">
LDLc: low-density lipoprotein cholesterol">
CAD: coronary artery disease">
apoB: apolipoprotein B">
PCSK9: proprotein convertase subtilisin/kexin type 9">
MEDPED: Make Early Diagnosis to Prevent Early Death">
DLCN: Dutch Lipid Clinic Network">
CU: carotid ultrasonography">
Lp(a): lipoprotein(a)">
BMI: body mass index">
CV: cardiovascular">
CVD: cardiovascular disease">
HDL: high-density lipoprotein">
MI: myocardial infarction
